# Erythema Multiforme: Unveiling the Unusual Effect of an Ayurvedic Medication ‘Jambavasa’ Used in the Treatment of Type 2 Diabetes Mellitus

**DOI:** 10.7759/cureus.43306

**Published:** 2023-08-10

**Authors:** Rinkle Gemnani, Satish Mahajan, Keyur Saboo, Utkarsh Pradeep, Aman Gupta

**Affiliations:** 1 Department of Medicine, Jawaharlal Nehru Medical College, Datta Meghe Institute of Higher Education and Research, Wardha, IND

**Keywords:** ayurvedic, case report, drug-induced, target lesions, viral infections, erythema multiforme (em)

## Abstract

Erythema multiforme (EM) is a rare immune-mediated condition that can manifest as cutaneous, mucosal, or both types of lesions. The target lesion, with concentric zones of color change, is a cutaneous feature that is typical of this illness. Despite the fact that a number of factors can lead to EM, the most common being Herpes simplex virus (HSV) infection, drug-induced EM is a rare entity. As disease severity and mucosal involvement vary across individuals, treatment should be optimized for each patient, considering the risk versus benefit ratio. To distinguish EM from other clinical imitators and to confirm the diagnosis, histopathologic tests and other laboratory procedures may be utilized. Our patient presented with symptoms suggestive of a viral infection, such as fever and rash, but the RTPCR report for various viral infections came out to be negative, hence indicative of the diagnosis of drug-induced erythema multiforme.

## Introduction

Erythema multiforme (EM) is a type of acute inflammatory dermatosis that is distinguished by the presence of various target lesions on the skin. Various conditions, including cancer, sarcoidosis, and drugs, have been linked to erythema multiforme; however, over 90% of cases can be attributed to infectious agents, the most common being herpes simplex virus in adults and mycoplasma pneumonia in children [1]. While it can have various etiologies, drug-induced erythema multiforme is a less common but important subtype that requires prompt identification and management. In roughly 10% of instances, the symptoms are linked to an adverse drug reaction to various drugs such as sulphonamides, anti-epileptic drugs, non-steroidal anti-inflammatory drugs, or antibiotics [1-2]. But rarely, ayurvedic medications can also lead to erythema multiforme.

Ayurveda is a traditional Indian medical system that has been used in India since 1500 BC. Its remedies are based on intricate herbal mixtures, minerals, and metals. The Ayurvedic medical system has always placed a strong emphasis on safe treatment, which includes minimizing symptoms while preventing the onset of new conditions. Unfavorable drug responses with Ayurvedic product ingredients, such as heavy metals and alkaloids, may cause adverse outcomes [3,4].

Although common in practice and Ayurvedic literature, these ADRs (adverse drug reactions) are not adequately addressed. Hence, it is important to emphasize this possibility so that both practitioners and customers will utilize Ayurvedic drugs with caution. TNF-alpha has been shown to contribute to the development of lesions in drug-induced erythema multiforme [1]. We present a rare case of drug-induced erythema multiforme in a patient with a positive history of hypertension and diabetes mellitus who had recently switched to ayurvedic supplements in the last 10 days.

## Case presentation

A 60-year-old female reported a three-day history of reddish to dusky rashes and fever to our emergency department. The patient initially developed painful erythematous rashes over the pulp of her fingers, palms, and soles, followed by the appearance of pruritic rashes over her upper extremities, upper back, and trunk that gradually progressed to pustules within 24 hours. The patient also complained of a high fever. The patient sought treatment from a local hospital for the above complaints and was prescribed oral medications in the form of tablets of paracetamol and levocetirizine. Despite the treatment, her symptoms persisted, leading her to seek care in our emergency department. The patient denied any contact with animals or individuals with fever or respiratory symptoms. The patient had a positive past medical history of hypertension and diabetes mellitus for the past six years, for which she was on tablets of telmisartan 40 mg once a day and metformin 500 mg twice a day earlier, but switched to the ayurvedic medication 'Jambavasa' for diabetes mellitus in the last 10 days.

**Figure 1:**
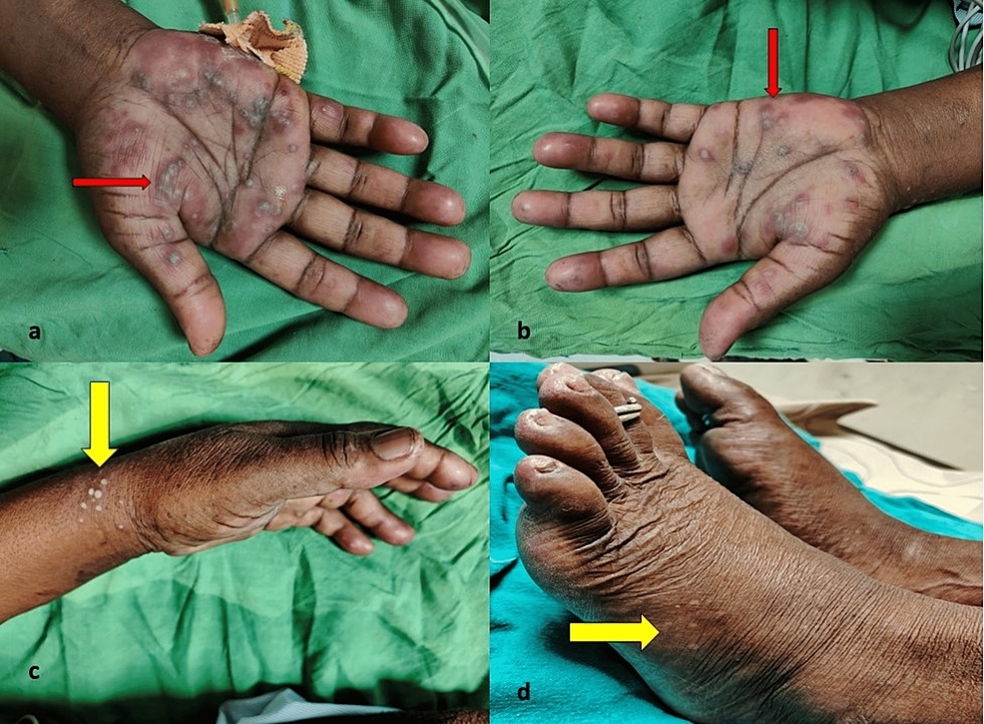
(a) and (b) Typical target lesions in palms (red arrow); (c) and (d) Pustules in the upper limb and lower limb (yellow arrow).

On presentation, the patient was dehydrated and agitated, with an ill appearance. Her pulse was 122 beats per minute, and her blood pressure was 90/60 mmHG. Her chest X-ray was normal. Upon physical examination, several 2-12 mm erythematous, urticarial, and targetoid papules were found. These papules had an annular wheal and a hyperpigmented core with purple to red duskiness. The lesions were observed bilaterally on the palms, dorsal hands, upper arms, upper back, trunk, and soles, without mucosal involvement, as shown in Figure 1. Neurologic, respiratory, and abdominal examinations were unremarkable. Consultations with dermatology and infectious disease specialists led to a provisional diagnosis of drug-induced erythema multiforme, considering the positive drug history of the ayurvedic medication 'Jambavasa' and characteristic skin lesions following which the ayurvedic medication was stopped.

**Figure 2:**
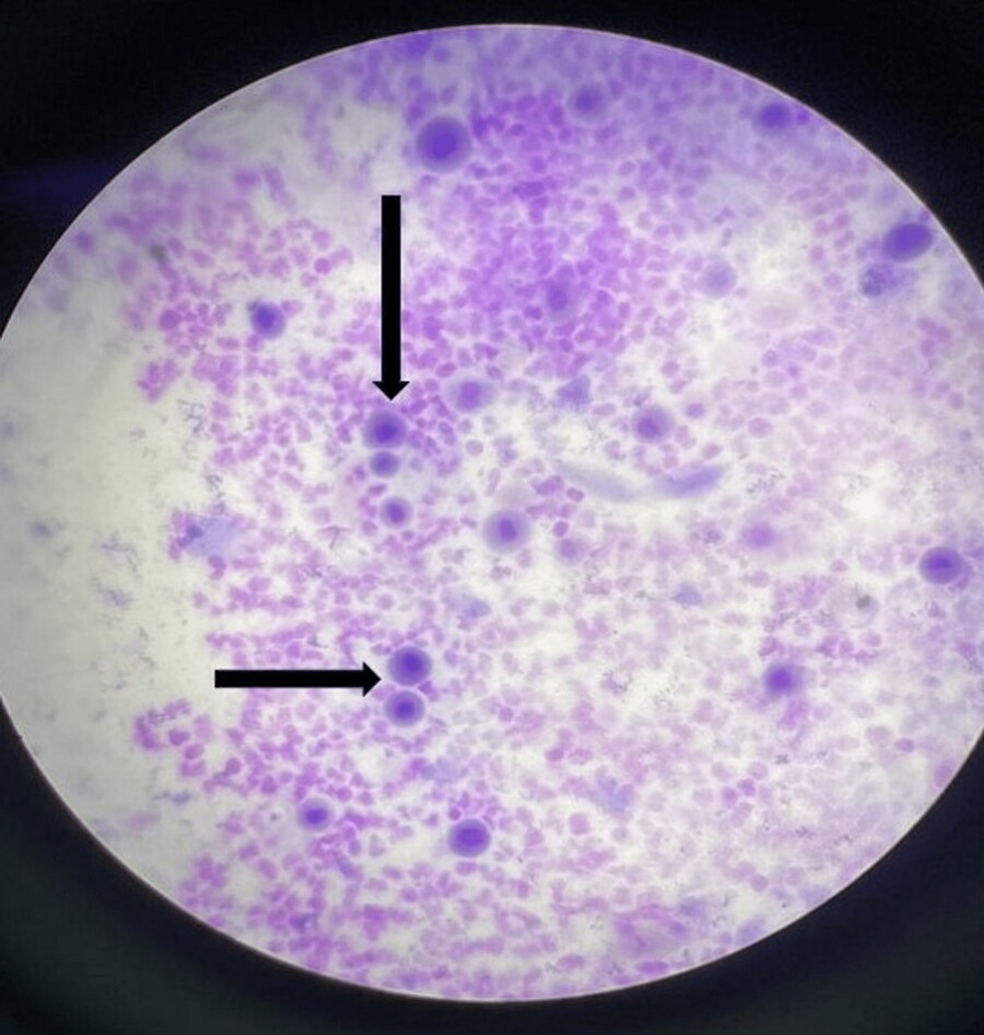
Tzanck smear shows numerous cytomorphologically normal acantholytic cells with inter-spread neutrophils (black arrow).

Laboratory workup included a complete blood count, a fever profile, bacterial and viral blood cultures, serology, and viral direct detection tests. The patient's complete blood count, kidney function test, and liver function test were within normal limits but had raised CRP levels of 159.68, as shown in Table 1. The fever profile was negative. Tests for herpes simplex virus (HSV) and human immunodeficiency virus (HIV) were negative. A tzanck smear was performed on palmar lesions, showing numerous acantholytic cells that were cytomorphologically normal with inter-spread neutrophils, as shown in Figure 2. To confirm blood samples, urine samples, and nasal and oropharyngeal swabs were sent for RTPCR for Herpes simplex, Varicella zoster, Monkeypox, and enterovirus, which were negative.

**Table 1:** Laboratory parameter of the patient with reference range.

Investigations	Patient	Reference Values
Haemoglobin	10.3 g/dl (low)	13–17 g/dl
Total leukocyte count	8900 /dl (normal)	4000–11,000/dl
Mean corpuscular volume	82 fl (normal)	83–101 fl
Platelet count	2,13,000 /dl (normal)	150,000–400,000/dl
Urea	30 mg/dL (normal)	19–42 mg/dl
Serum creatinine	0.9 mg/dL (normal)	0.5–1.2 mg/dl
Sodium	132 mmol/L (low)	137–145 mmol/L
Potassium	4.5 mmol/l (normal)	3.5–5.1 mmol/L
Albumin	3.6 g/dl (normal)	3.5–5.0 g/dl
Aspartate aminotransferase	39 U/L (normal)	<50 U/L
Alanine aminotransferase	50 U/L (normal)	17–59 U/L
Total bilirubin	1.0 mg/dl (normal)	0.2–1.3 mg/dl
C-reactive protein	159.6 mg/dl (raised)	<6 mg/dl
Activated partial thromboplastin time	32.0 (high)	29.5–31.0
Prothrombin time	17.0 (high)	11.9–13
International normalized ratio	1.01 (normal)	0.9–1.4

**Figure 3:**
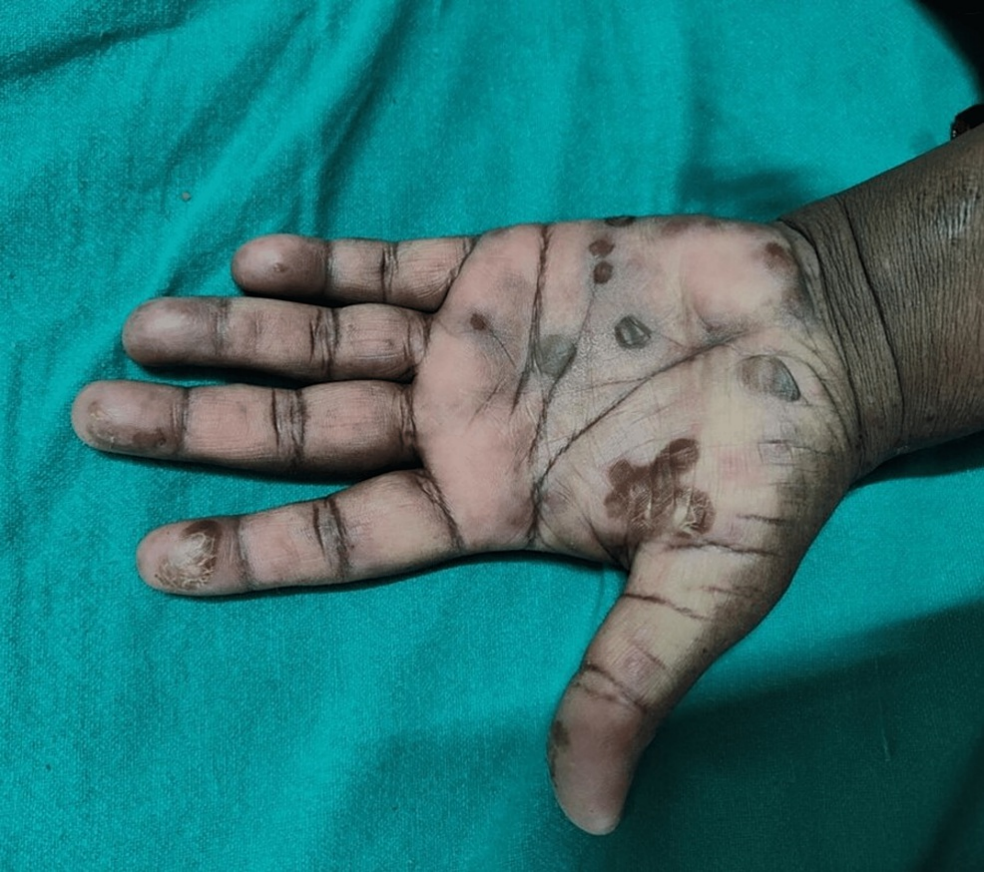
Resolving lesions of erythema multiforme.

The patient was initially started on injection acyclovir, which was discontinued following negative laboratory results, after which the patient was commenced on systemic corticosteroid tablets (prednisolone 20 mg twice a day) along with ointment triamcinolone acetonide 0.1% three times a day. After 48 hours of initiating the treatment, the patient improved clinically and the fever subsided. After 15 days, the systemic steroids were tapered by 10 mg per week until a maintenance dose of 5 mg was reached as the lesions clinically regressed, as shown in Figure 3.

## Discussion

Erythema multiforme can be classified into minor, major, and persistent variants that can present as either typical or atypical skin lesions. The hallmark feature of this condition is the presence of targetoid lesions on the extensor surfaces of the acral extremities [5]. These lesions consist of a dusky central blister, a dark red inflammatory zone, a pale edematous ring, and an erythematous halo around the lesion. The atypical lesions are edematous and elevated, with two color-changing zones and an ill-defined border. Typical or atypical presentations may involve the face, neck, palms, soles, flexor surfaces, and trunk. In addition to cutaneous lesions, lesions can also appear in the mucous membranes of the oral, ocular, or vaginal mucosa. According to estimates, oral involvement affects 25-60% of erythema multiforme individuals. Erythema multiforme is further distinguished into major and minor types based on the severity of the illness and the location of the lesions. Although both variations have many of the same traits, erythema multiforme major is distinguished by lesions that involve one or more mucosal membranes and is linked to a more severe presentation. Erythema multiforme minor, in contrast, frequently manifests with fewer or no mucosal membrane symptoms and milder cutaneous signs [1].

Lesions of erythema multiforme often develop over three to five days and disappear in one to two weeks. However, the resolution could take up to six weeks or more for severe cases of erythema multiforme involving the mucosal membranes. On average, these episodes may occur up to six times per year, lasting between six and ten years. Recurrent erythema multiforme, a subtype of the condition, has been linked to a herpes simplex virus infection. In a few cases, lesions develop continuously over an extended period (more than a year) without interruption. This illness is referred to as persistent erythema multiforme, and viral infections may be linked to it [1].

The most frequent cutaneous ADRs to any type of drug are rashes, which often develop within the first week of drug administration. Many allopathic drugs, including penicillin, cephalosporins, drugs of the sulfa group, allopurinol, and NSAIDs, have been shown to cause severe cutaneous reactions, including Stevens-Johnson Syndrome (SJS), toxic epidermal necrolysis (TEN), acute generalized exanthematous pustulosis (AGEP), and drug reactions with eosinophilia and systemic symptoms [6,7]. However, there are not many studies in the literature that address Ayurvedic medicines as a potential contributor to severe cutaneous adverse reactions. So, to solve this problem scientifically, pharmacovigilance must be implemented in Ayurveda [3].

Ayurvedic multi-herb formulations are typically offered as a combination of several plant components that play an essential role in the cure of ailments. The Ayurvedic medication ‘Jambavasa’ is one such polyherbal medication containing jamun (Eugenia jambolana, Myrtaceae), neem (Azadirachta indica, Meliaceae), methi Beej (seed of Trigonella foenum-graecum, Apiaceae), Karela (Momordica charantia, Cucurbitaceae), shudha Shilajeet, Gudmar (Gymnema sylvestre, Sclepiadaceae), Triphala (Terminalia belerica, Combretaceae; Terminalia chebula, Combretaceae; Triwang bhasma [Shuddha Naga (purified lead), Shuddha Vanga (purified tin), Shuddha Yashada (purified zinc)], Dhataki (Woodfordia fruticosa, Lythraceae), etc., are used in the treatment of diabetes mellitus by targeting free radical damage and providing an anti-oxidant effect. Although the exact pathogenesis is not known, according to the literature, cutaneous skin reactions, like erythema multiforme in our case, are caused by the reaction between the components of these polyherbal medications [8,9]. Anjal et al. have also reported cutaneous reactions in the form of generalized skin rashes after oral administration of other ayurvedic polyherbal medications [3].

Erythema multiforme cannot be diagnosed using laboratory procedures; however, serologic testing for autoantibodies, such as antinuclear antibodies, should be taken into consideration if autoimmune blistering illnesses are included in the differential diagnosis [10]. As there are no specific diagnostic tests, diagnosis is mainly clinical and can be supported by biopsy, if necessary, mainly in atypical variants.

Erythema multiforme has a self-limited clinical course; however, patients with moderate to severe disease with pain and mucosal involvement have reported improvement with strong systemic glucocorticoids. Antiviral drug therapy has proven to be an effective prophylaxis in patients with recurrent erythema multiforme. Patients with a clear association between herpes simplex infection and erythema multiforme have the best response to therapy [2].

## Conclusions

Drug-induced Erythema multiforme is an uncommon variant of Erythema multiforme, as Erythema multiforme is frequently caused by HSV infections and rarely as a result of adverse drug reactions. Even though a primary attack of drug-induced EM can cause mild symptoms, subsequent attacks can produce more severe forms of EM (EM minor and EM major) involving the skin and mucous membrane. As a result, it is critical to distinguish drug-induced EM from other vesiculobullous diseases. As there is no specific diagnostic test, early recognition and prompt disease diagnosis are critical to initiating appropriate treatment.
